# TILs Immunophenotype in Breast Cancer Predicts Local Failure and Overall Survival: Analysis in a Large Radiotherapy Trial with Long-Term Follow-Up

**DOI:** 10.3390/cancers12092365

**Published:** 2020-08-21

**Authors:** Ewan Millar, Lois Browne, Iveta Slapetova, Fei Shang, Yuqi Ren, Rachel Bradshaw, Heather Ann Brauer, Sandra O’Toole, Julia Beretov, Renee Whan, Peter H. Graham

**Affiliations:** 1 Department of Anatomical Pathology, NSW Health Pathology, St George Hospital, Kogarah, NSW 2217, Australia; julia.beretov@health.nsw.gov.au; 2 Faculty of Medicine, St George & Sutherland Clinical School, University of New South Wales Sydney, Kensington, NSW 2052, Australia; peter.graham@health.nsw.gov.au; 3 Faculty of Medicine & Health Sciences, Sydney Western University, Campbelltown, NSW 2560, Australia; 4 Cancer Care Centre, St George Hospital, Kogarah, NSW 2217, Australia; lbrowne@tpg.com.au; 5 Biomedical Imaging Facility, Mark Wainwright Analytical Centre, University of New South Wales Sydney, Kensington, NSW 2052, Australia; i.slapetova@unsw.edu.au (I.S.); f.shang@unsw.edu.au (F.S.); r.whan@unsw.edu.au (R.W.); 6 NanoString Technologies Inc., Seattle, WA 98109, USA; yren@nanostring.com (Y.R.); rbradshaw@nanostring.com (R.B.); habrauer@nanostring.com (H.A.B.); 7 Department of Anatomical Pathology, NSW Health Pathology, Royal Prince Alfred Hospital, Camperdown, NSW 2217, Australia; sandra.otoole@health.nsw.gov.au; 8 Garvan Institute of Medical Research, Victoria Street, Darlinghurst, NSW 2010, Australia; 9 Faculty of Medicine, University of Sydney, Camperdown, NSW 2050, Australia

**Keywords:** tumour infiltrating lymphocytes (TILs), immunophenotype, prognosis, breast cancer

## Abstract

Aim: To determine the prognostic significance of the immunophenotype of tumour-infiltrating lymphocytes (TILs) within a cohort of breast cancer patients with long-term follow-up. Methods: Multiplexed immunofluorescence and automated image analysis were used to assess the expression of CD3, CD8, CD20, CD68, Fox P3, PD-1 and PD-L1 in a clinical trial of local excision and radiotherapy randomised to a cavity boost or not (*n* = 485, median follow-up 16 years). Kaplan–Meier and Cox multivariate analysis (MVA) methodology were used to ascertain relationships with local recurrence (LR), overall survival (OS) and disease-free survival (DFS). NanoString BC360 gene expression panel was applied to a subset of luminal patients to identify pathways associated with LR. Results: LR was predicted by low CD8 in MVA in the whole cohort (HR 2.34, CI 1.4–4.02, *p* = 0.002) and luminal tumours (HR 2.19, CI 1.23–3.92, *p* = 0.008) with associations with increased stromal components, decreased Tregs (FoxP3), inflammatory chemokines and SOX2. Poor OS was associated with low CD20 in the whole cohort (HR 1.73, CI 1.2–2.4, *p* = 0.002) and luminal tumours on MVA and low PD-L1 in triple-negative cancer (HR 3.44, CI 1.5–7, *p* = 0.003). Conclusions: Immunophenotype adds further prognostic data to help further stratify risk of LR and OS even in TILs low-luminal tumours.

## 1. Background

Tumour-infiltrating lymphocytes (TILs) are currently considered an important emerging prognostic factor, with high TILs in triple-negative breast cancer (TNBC) associated with improved prognosis in postneoadjuvant-treated (>60%) [1], adjuvant-treated early disease [2] or untreated disease [3] (both >30%). Although the TILs Working Group (TWG) published guidelines on interpretation and scoring criteria [4,5] with good reproducibility, these do not account for immune subtypes, and the relevance of TILs is not clearly established for luminal tumours. Numerous studies assessed the prognostic significance of T-cell and B-cell cell markers, predominantly in TNBC or HER2-positive cancer, with most demonstrating improved survival with increased immune cell density; however, less data exists regarding associations with outcome in luminal tumours and local recurrence (LR) in the breast. Therefore, identifying immune-related prognostic information in estrogen receptor positive (ER+) disease, which comprises 75–80% of all tumours, may add value to clinical decision-making and risk assessment of LR. 

Given the powerful capacity of multiplexing to reveal the spatial expression of several immuno-oncology markers, we aimed to describe the associations of T-cell, B-cell, histiocytic and programmed cell death phenotypic markers (CD3, CD8, CD20, CD68, Fox P3, PD-1 and PD-L1) using automated image analysis in a large, well-characterised breast cancer cohort (all subtypes), with long-term clinical follow-up (median 16 years). Unlocking potentially useful spatial information in the complex tissue microenvironment (TME) may help to further guide clinical decision-making. Manual counting of thousands of cells, differentially stained with multiple coloured fluorescent markers, is not a practical option for the detailed assessment of large, retrospective research cohorts. Therefore, the development and evaluation of automated image analysis methods with machine learning segmentation algorithms is more applicable to next-generation biomarker development.

## 2. Materials and Methods

### 2.1. Clinical and Pathological Details

**Figure 1:**
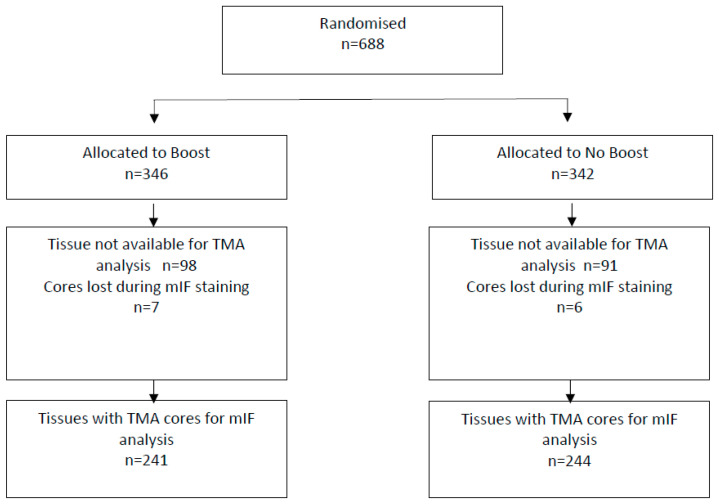
CONSORT flow diagram. The trial recruited from three main centres (St George, Wollongong and Liverpool Hospitals). Although the total number of patients assessed for eligibility and excluded for all centres is not known, these data are available for the main recruiting centre at St George Hospital, which contributed the majority of patients participating in the trial *n* = 546.

The study cohort consisted of 498 invasive breast cancers of 688 patients originally recruited to the St George Breast Boost study (1998–2003), 629 of which had invasive carcinoma (59 had ductal carcinoma in-situ (DCIS)). All patients received wide local excision with whole breast irradiation (45Gy with boost; 50Gy if no boost in 25 fractions), randomised to a cavity boost (16Gy in 8 fractions) or not (ClinicalTrials.gov NCT00138814). A CONSORT diagram of the cohort is provided in Figure 1. This study recruited all subtypes of breast cancer stages Tis-3, N0-3, M0, with a current median follow-up of 16 years (details as previously published [6,7]; survival data updated to December 2019, Table S1), 45% received endocrine therapy, 24% received chemotherapy (CMF or AC) and 9% received both. No patients received Herceptin or other anti-HER2 therapies. All tissue microarrays (TMAs) were constructed using 3 × 1 mm cores per patient tumour (sampled from the tumour peripheral edge) using MTA-1 Manual Tissue Arrayer (Beecher Instruments, Inc., Sun Prairie, WI, USA), which were stained and manually scored using immunohistochemistry (IHC) for ER, PR, Ki67 and HER2 FISH, as previously described [6,7]. Tumours were typed histologically using WHO criteria [8], graded using the Nottingham system [9] and classified for intrinsic subtype according to the following definitions [10,11,12,13] as Luminal A (LA): ER+ (≥1%), PR > 20%, HER2, Ki67 < 14%; Luminal B (LB): ER+, PR 20% and/or HER2+ and/or Ki67 ≥ 14%; HER2-enriched: ER− (<1%), PR− (<1%), HER2-amplified; triple-negative (TNBC) ER− (<1%), PR− (<1%), HER2−. TILs were scored from H/E sections of TMAs of the cohort, according to published guidelines [5]. The Reporting Recommendations for Tumour Marker Prognostic Studies (REMARK) criteria were followed throughout [14].

### 2.2. Multiplexed Immunofluorescence (mIF)

**Figure 2:**
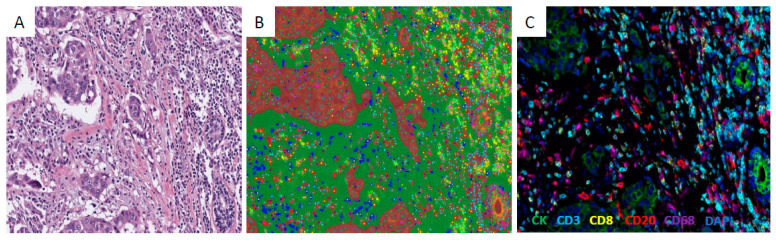
(**A**) Invasive ductal carcinoma with stromal tumour-infiltrating lymphocytes (TILs). (H/E ×200); (**B**) tumour epithelium and stromal segmentation (×200); (**C**) multiplexed immunofluorescence (×400).

Multiplexed Immunofluorescence staining was manually performed on 4-μM-thick, formalin-fixed, paraffin embedded (FFPE) tissue microarray (TMA) sections, using Opal^TM^ 7 Multiplex reagents (PerkinElmer, Inc., Waltham, MA, USA), as per the manufacturer’s instructions (see File S1) and as previously described in other studies [15,16,17]. Staining was performed as 2 separate panels with DAPI as a nuclear counterstain: panel 1 (Figure 2A–C): panCK (PerkinElmer, OP7LT4001K) opal 540, CD3 (DAKO Agilent Santa Clara, CA, USA, M7254) opal 570; CD8 (PerkinElmer, OP7LT4001K) opal 520, CD20 (PerkinElmer, OP7LT4001K) opal 620; CD68 (PerkinElmer, OP7LT4001K) opal 650; panel 2: panCK (PerkinElmer, OP7LT4001K) opal 540; CD3 (DAKO, M7254) opal 570; PD-1 (Abcam, Cambridge, MA, USA ab137132) opal 650; PD-L1 (Cell Signalling, Danvers, MA, USA #13684) opal 520; FoxP3 (PerkinElmer, OP7LT4001K) opal 620, with DAPI nuclear counterstain. Fluorophore-stained slides were scanned and images were acquired using the Vectra Polaris platform (PerkinElmer Inc.). PanCK was used to perform segmentation of tumour epithelium and stroma using a trained segmentation algorithm within inForm^®^ image analysis software version 2.3 (PerkinElmer Inc.), supervised by a pathologist. Spectral unmixing provided automated cell count scores for each phenotypic marker per 1 mM diameter core (×200 power objective; 0.785 mM^2^) in the stromal and epithelial compartments. CD3 cell counts were averaged across both panels. Scores for the stromal compartment only were used for all statistical analyses.

### 2.3. PAM50, NanoString ROR Score and BC360 Gene Expression Panel in Luminal Cancer with Local Recurrence

The BC360 panel contains 776 breast cancer related and immuno-oncology genes and 48 signatures, and includes the PAM50 intrinsic subtype and Prosigna^©^ risk of recurrence (ROR) score. mRNA was extracted from FFPE blocks of 50 luminal A and B patients (classified by IHC), 25 with local recurrence and 25 grade-, size- and cavity boost-matched patients without recurrence with sufficient tumour within residual paraffin blocks, using the RNA easy FFPE extraction kit (Qiagen GmbH, Hilden, Germany), according to the manufacturer’s instructions. The assay was run on the nCounter platform at UNSW Sydney. 

### 2.4. Statistical and Bioinformatics Analyses

Associations of cell count scores of immune cell markers with IHC intrinsic subtype were assessed using the Kruskal–Wallis test; *p* < 0.05 was considered significant. Comparisons between intrinsic subtype groups were made using the Mann–Whitney–Wilcoxon test. Associations of immune markers with clinicopathological features were performed using the X^2^ test using the median value of each immune marker as the cut point for high or low values. Time to event outcomes (LR: any recurrence (invasive or noninvasive) in the treated breast; OS: time from randomization to death from any cause; DFS: time to LR, LRR or distant metastases (or earliest event if more than 1 event)) were assessed using Cox proportional hazards for univariate and multivariate analyses, where *p* < 0.05 was considered significant. Survival estimates were displayed using Kaplan–Meier analyses. All analyses were performed using STATA V11 (StataCorp LLC, College Station, TX, USA).

Bioinformatics data analysis was performed by NanoString Technologies, Inc (Seattle, CA, USA). Genes were normalised using the ratio of expression value to the geometric mean of a set of housekeeping genes and then further normalised using a ratio of the housekeeper-normalised data to a panel standard containing known concentrations of all probes. A final Log(2) transformation was performed on the normalised gene counts. Differential expression was fit on a per signature basis using a liner model. The statistical model used the signature score as the dependent variable and fit LR as a fixed effect. 

## 3. Results

### 3.1. Associations of TILs Immunophenotype with Intrinsic Subtype

The whole cohort consisted of a total of *n* = 498. Due to antigen retrieval and processing of the TMAs, there was loss of cores for 13 cases, resulting in data available for 485 tumours: LumA 309, LumB 96, TNBC 67, and HER2 13 (see CONSORT flow diagram, Figure 1).

**Figure 3:**
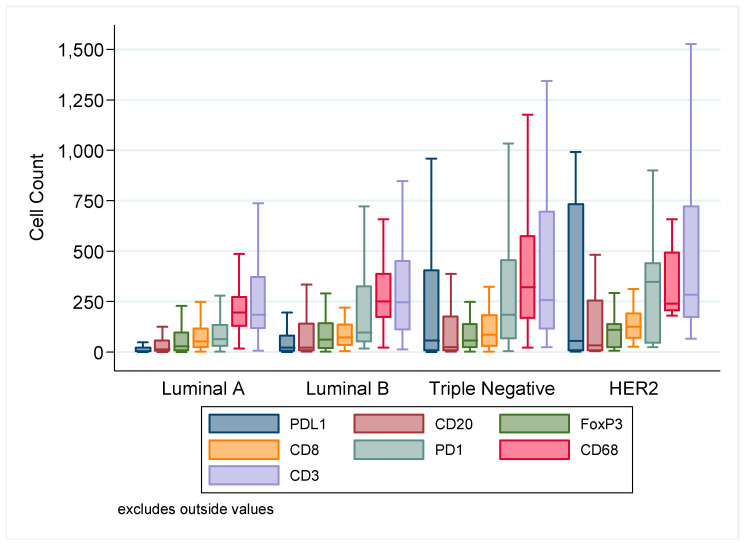
Immune cell counts according to intrinsic subtype of breast cancer. There is a significant difference in expression for all markers across each subtype (CD3, *p* = 0.026; CD8, *p* = 0.017; CD20, *p* = 0.006; CD68, *p* < 0.001; PD-1, *p* < 0.001; PD-L1, *p* < 0.001; Kruskall–Wallis test).

The composition of immunophenotype of TILs within intrinsic subtypes is presented in Figure 3. Based on the average cells counts, CD3 was the most numerous cell type, with a mean of 320, followed by CD68 269, PD-1 179, CD20 135, CD8 108, PD-L1 104 and FoxP3 100. TNBC and HER2 tended to have higher numbers of immune cells present compared to LumA and LumB. The associations of immune cell counts with intrinsic subtype are presented in Table S2. Each immune marker was significantly different across each intrinsic subtype (Kruskall–Wallis test). The median value for the cell counts of each immune marker was used as the cut point to determine high or low expression levels: CD3: 200; CD8: 61; CD20: 15; CD68: 213; FoxP3: 35; PD1: 78; PDL1: 10. For the whole cohort, the sTILs average was 10% with a median of 5% and a range of 0–90%.

The associations of immune markers and sTILs with clinicopathological features are presented in Table S3A,B. In summary, high CD3, CD20, FoxP3, PD-1, PD-L1 and sTILs correlated with grade 3, ER-negative, HER2-positive and age of <50, CD8 correlated with grade 3 and age of <50 and CD68 with grade 3 and ER-negative status.

### 3.2. Local Recurrence

**Figure 4:**
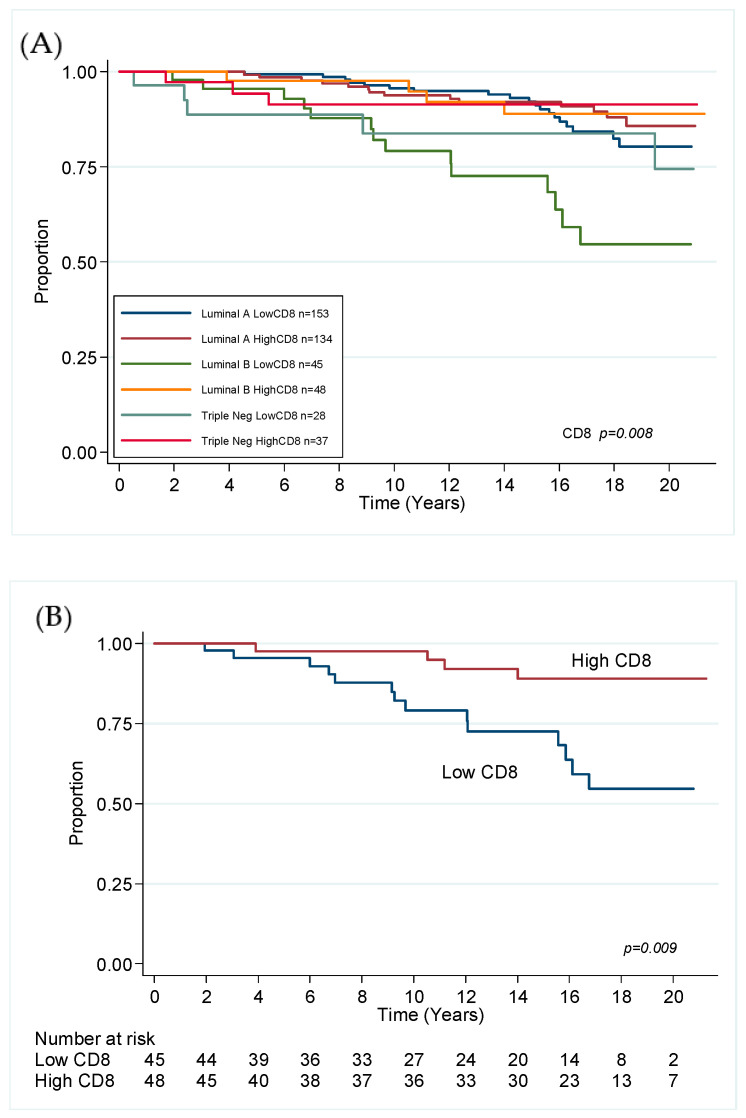
(**A**) Kaplan–Meier estimates for local recurrence in the whole cohort and (**B**) in luminal B cancer, stratified by CD8 expression.

Cox proportional hazard modelling of immune markers and clinicopathological features in univariate and multivariate analyses (MVA) are presented in Table 1 for the whole cohort. There were 60 events. Low CD8 was predictive of LR in the whole cohort (HR 2.3, CI 1.4–4.0, *p* = 0.002) in a final model adjusted for grade, margin status and boost status. Figure 4A displays survival curves by molecular subtype stratified by CD8 status, with LB in Figure 4B. This result was also observed in an MVA of luminal tumours only, making up 84% of the cohort, where the final resolved model included CD8 (HR 2.2, CI 1.2–3.9, *p* = 0.008) age of <50, margin status and radiotherapy boost (Table S4). MVA of TNBC showed none of the immune markers to be prognostically significant for LR, only cavity boost status (Table S5; HR 9.9; 1.2–80.9, *p* = 0.032).

**Figure 5:**
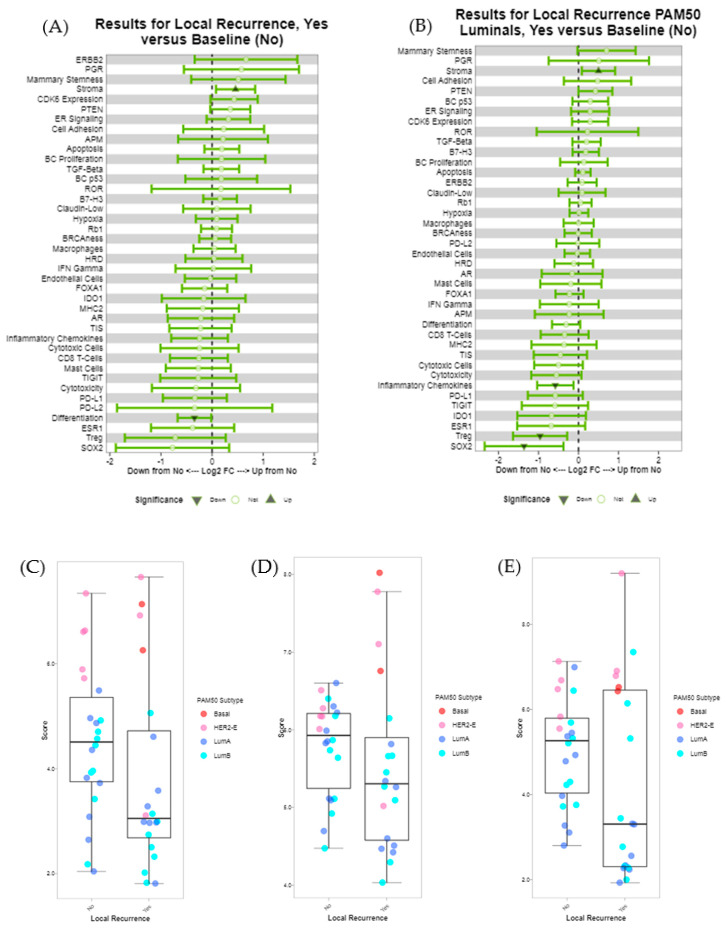
Forest plot of gene expression signatures using the NanoString BC360 panel. (**A**) Forest plot of BC360 signatures for IHC luminal cases. (**B**) Forest plot of BC360 signatures for PAM50 luminal cases. (**C**) Boxplot of Treg scores for all IHC luminal cases, coloured by PAM50 subtype. (**D**) Boxplot of inflammatory chemokines for all IHC luminal cases, coloured by PAM50 subtype. (**E**) Boxplot of SOX2 for all IHC luminal cases, coloured by PAM50 subtype.

To further investigate the immune microenvironment and breast cancer molecular features associated with LR in luminal tumours, we performed mRNA expression profiling using the NanoString BC360 panel in 50 tumours classified as luminal A or B by IHC. In all samples, the mRNA was heavily degraded but passed internal QC prior to data analysis. Of the 50 samples, 42 passed the additional PAM50 QC and had subtypes classified as LumA 16, LumB 16, HER2-enriched 8 and basal 2. The heatmap of gene expression is presented in Figure S1. Within the group of 50 luminal tumours, LR was associated with increased stromal abundance and decreased differentiation. Several immune signatures were trending lower in cases with local recurrence, including PD-L1, TIGIT, IDO1 and the tumour inflammation signature (TIS), however, these did not reach statistical significance. Within the tumours classified as luminal A or B by PAM50, LR was associated with increased stromal abundance and decreased inflammatory chemokines, Treg (FoxP3) and SOX2 (Figure 5A–E), but was not associated with risk of recurrence (ROR) score or PAM50 subtype. 

### 3.3. Overall Survival

**Figure 6:**
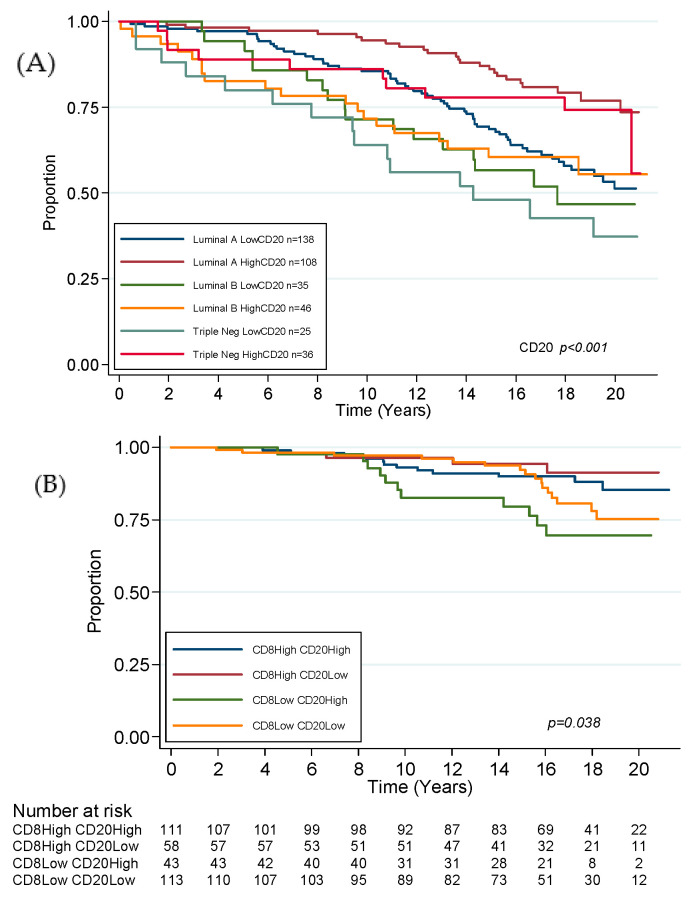

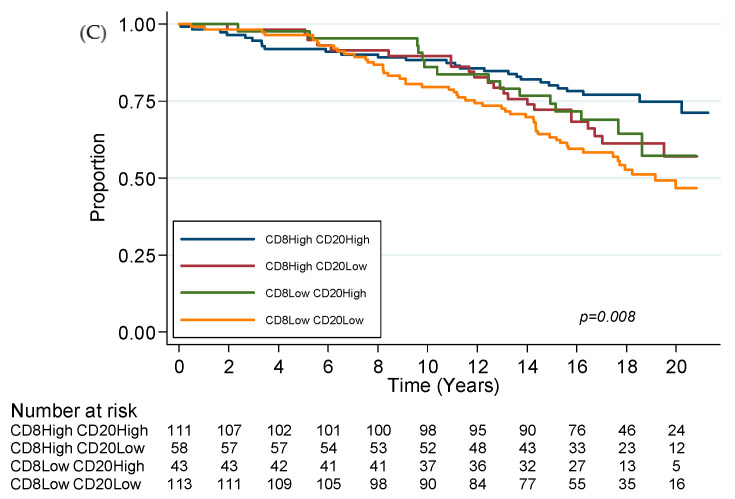
(**A**) Kaplan–Meier estimates for overall survival in the whole cohort stratified by CD20 expression; combined CD8 and CD20 expression in luminal-only tumours for (**B**) local recurrence and (**C**) overall survival.

In the whole cohort, low CD8, CD20, PD-1, FoxP3 and sTILs were associated with poor OS in univariate analysis. MVA demonstrated CD20 to be predictive of poor OS (HR 1.7, CI 1.2–2.4, *p* = 0.002) in a final model adjusted for lymph node status and age (Table 2). Figure 6A displays survival curves by molecular subtype, stratified by CD20 status. For luminal tumours only, low CD8, CD20 and PD-1 were associated with poor OS in univariate analysis, with CD20 shown to be significant in MVA (HR 1.7, 1.1–2.4, *p* = 0.008, Table S6). 

In TNBC, low CD20, Fox P3, PD-L1 and PD-1 were associated with poor OS in univariate analysis, with PD-L1 (HR 3.4, 1.5–7.73, *p* = 0.003) predictive of poor outcome in MVA in a final model including lymph node status (Table S7).

### 3.4. Combined CD8 CD20 Status and Outcome

Given the significance of CD8 and CD20 in predicting outcome in our cohort and in published literature, we performed further exploratory analyses by dividing tumours into four groups based on CD8 and CD20 high and low expression. For LR, this classification was significant in the whole cohort (*p* = 0.009) and luminal tumours (*p* = 0.038, Figure 6B), but not in TNBC. This demonstrated that the highest risk was seen with CD8-low, CD20-high expression, which over the whole cohort was over three times the hazard of LR compared to CD8–CD20-double-high tumours (HR 3.4, CI 1.6–7.1, *p* = 0.001), and more than twice the hazard in luminal-only tumours (HR 2.7, CI 1.2–6.2, *p* = 0.016).

For OS, double-low-CD8–CD20 status identified a poor prognostic group in the whole cohort with more than twice the risk of death compared to CD8–CD20-double-high tumours (HR 2.3, CI 1.5–3.4, *p* < 0.001). This was also observed in luminal-only tumours (HR 2.2, CI 1.4–3.5, *p* = 0.001, Figure 6C). For TNBC, CD20-low, CD8-high identified a statistically significant poor prognostic group, with double-low status not quite significant. Further analyses were performed for combined CD8–PD-L1 and CD20–PD-L1, which were not significant with any tumour group for LR. However, associations were identified for OS in the whole cohort, luminal and TNBC.

### 3.5. Disease-Free Survival (DFS)

In the whole cohort, MVA demonstrated low CD8 to be predictive of disease recurrence (HR 1.6, CI 1.1–2.3, *p* = 0.015) in a final model adjusted for lymph node status, endocrine therapy, tumour size and margin status (Table S8). MVA for luminal tumours showed low CD8 to again be predictive of recurrence (HR 1.7, CI 1.1–2.6, *p* = 0.011, Table S9), whilst in triple-negative cancers, low PD-1 (HR 8.6, CI 2.3–31.5, *p* = 0.001) predicted recurrence in a final model adjusted for lymph node status, lymphovascular invasion and radiotherapy boost (Table S10).

## 4. Discussion

This study utilised multiplexed immunofluorescence and automated image analysis to characterise the immunophenotype of TILs in the tumour microenvironment (TME) in a well-characterised cohort of breast cancer patients with long-term follow-up. This cohort was recruited to a randomised trial of a radiotherapy boost to the cavity in conservatively treated breast cancer, and therefore provided the opportunity to examine the role of immunophenotype of TILs and associations with LR in addition to OS. Significantly, we identified low stromal CD8 to be an independent predictor of LR in the whole cohort and in luminal breast cancer. These data are of interest, as they suggest a role for CD8 T-cells in synergising with radiotherapy (RT) to improve antitumour immunity in ER+ tumours, which have relatively low numbers of TILs present, independent of treatment, tumour grade, margin status or a cavity boost. We were unable to identify any association of immune cells with LR in TNBC, although this may reflect the small number of cases in the cohort with relatively few events (67 tumours and 8 events).

Radiotherapy plays a central role in optimising local control in conservatively treated breast cancer, with an EBCTCG meta-analysis demonstrating the addition of RT to local excision reduces LR by two-thirds (8.8% vs. 27.2% local recurrence by year 10) [18]. Whilst the mechanism of action of RT has long been considered to be DNA damage via double strand breaks and generation of reactive oxygen species (ROS), more recently renewed interest was sparked regarding its potential as an immune stimulant. The induction of immune death by RT is thought to result in the release of tumour-associated antigens and DNA into the local TME, resulting in increased interferon type 1 (IFN-1) via the cGAS/STING pathway, promoting antigen presentation by BATF3-dendritic cells to prime the immune response and driving the recruitment and expansion of local CD8+ T-cells [19]. This process is considered to be a mechanism for “abscopal effects”, seen rarely in distant metastatic deposits, and supports the clinical observation that local RT is known to reduce annual mortality rates by 13.2% [18]. Several clinical trials of immune checkpoint blockade (ICB) plus RT are underway, with hypofractionated schedules observed to be more effective at immune induction than a single large dose (reviewed in [20]). Our data support these observations and significantly identify CD8 as a prognostic biomarker independent of treatment modality (endocrine, chemotherapy, radiotherapy) and margin status. We also provide further evidence to highlight the importance of CD8 T-cells and CD20 B-cells by further stratifying the risk of recurrence where low-CD8, high-CD20 tumours exhibited more than twice the risk of LR of double-high tumours (HR 2.7, CI 1.2–6.2, *p* = 0.016). Current clinical risk prediction for LR in the breast includes online algorithms such as IBTR! (https://www.evidencio.com/models/show/1385), which combines clinical and pathological features to provide a risk estimation. Our data suggest that an immune component could be built into such a model to potentially improve risk stratification for ER+ cancer.

Interrogation of a small subset of luminal tumours using the NanoString BC360 mRNA panel showed LR to be associated with stromal predominance in both the IHC luminal cohort and the PAM50 luminal cohort. However, decreased Treg, inflammatory chemokines and SOX2 signatures were associated with LR when examining the PAM50 luminal cohort only. These signatures were trending lower in the IHC luminal cohort but were not statistically significant. In the PAM50 luminal cohort, the subset of immune signatures (PD-L1, TIGIT, IDO1, TIS) trending lower in LR may have been linked to a suppressive immune response among recurrent luminal tumours. Additionally, a decrease in FoxP3 (Treg signature) was also associated with poor overall survival in both luminal and TNBC samples by IHC, thus indicating a role for decreased immune regulation associated with poor survival.

For OS, CD20 was independently prognostic in the whole cohort and also in luminal tumours. The combination of CD8 and CD20 was again utilised to create a predictive classifier, where double-high status predicted good prognosis and double-low predicted poor prognosis. These data support the findings of another study, where CD8 and CD20 were both independently prognostic of breast cancer-specific survival (BCSS) [21]. These findings support the cooperative role that B-cells and T-cells have to produce a more potent immune response. T-cells are the most abundant immune cell in the TME and exist as several different subtypes characterised by distinct immunophenotypes, with a stepwise increase in TILs from normal to DCIS to IDC [22]. T-cells in breast cancer were extensively characterised with a meta-analysis of over 12,000 breast tumours demonstrating high levels of CD8, where T-cells were associated with improved BCSS in TNBC, HER2+ and ER+/HER2+ tumours but not ER+ HER2− [23]. Recent data also indicated how immunophenotype relates to genomic abnormalities, where CD8+ T-cells were found to be more abundant with PI3KCA mutations in ER+ breast cancer [24], which may partially explain the findings of higher TILs in poorly responsive neoadjuvant aromatase inhibition (AI) in ER+ [25]. ER signalling may modify the immune response via IFN-γ downregulation of HLA-class I and II expression and type 1 cytokine expression, resulting in downregulation of T-cell tumour cell killing [26]. Increased TILs were described with reduced proliferative suppression in neoadjuvant, endocrine-treated, postmenopausal, ER+ breast cancer [25] with increased levels of immune checkpoint components IDO1, PD-1 and LAG3 described with AI-resistant proliferation in luminal B cancer [27]. Additionally, increased cell geospatial clustering was shown to correlate with poor recurrence-free survival in ER+ cancer [28]. Better characterisation of these associations may allow improved selection of patients for therapeutic decision-making, potentially through artificial intelligence approaches to screening of tumour H/E images or multiplexed mIF/IHC, for underlying genomic abnormalities of clinical relevance [29]. B-cells are also critically important and often make up a moderate proportion of TIL infiltrates. Of seven published breast cancer studies, five found a positive prognostic effect and two were not significant (reviewed in [30,31]). B-cells are capable of stimulating tumour-specific T-cells via IL-2, IL-4, IFN-γ and TNFα and by acting as specialised antigen presenting cells (APCs), differentiating into plasma cells, producing tumour-specific immunoglobulin, activating complement and promoting antibody-dependent, cell-mediated cytotoxicity (ADCC) [32]. However, a suppressive function was also described, where regulatory B-cells (Bregs) inhibit CD8+ T-cell-generation of potent immune responses via IL-10, IL-35 and TGF-β [33]. More recently, B-cells and tertiary lymphoid structures (TLS) were shown to correlate with more potent responses to immune checkpoint blockade (ICB) in melanoma [34]. TILS are relatively uncommon in most breast cancer subtypes other than in TNBC [35]. Significantly, CD20+ B-cells and PD-L1 were found to be of prognostic significance in inflammatory breast cancer treated with neoadjuvant chemotherapy [36], with high CD20 also associated with a pathological complete response (pCR) in neoadjuvant chemotherapy, but not CD3 or CD8 [37]. 

Although we found an association of poor outcome of low FoxP3 with LR in luminal tumours, this was not significant in MVA. Other studies found that high levels of FOXP3+ TILs predicted poor prognosis in luminal tumours [38], with an association of poor response to neoadjuvant letrozole in post treatment biopsies [39]. However, definitively dissecting the role of Treg requires double labelling with CD4 and FOXP3, as FOXP3 is also expressed by activated CD8+ T-cells [40]. 

Whilst the scoring of TILs was shown to be reproducible, its assessment is semiquantitative and subject to some interobserver error and does not include subtype estimation or their contributions to the immune infiltrate. Multiplexed immunofluorescence or immunohistochemistry could provide these data in a quantitative fashion and allow us to further tease prognostic data from such studies. With automation comes the potential for the development of machine learning algorithms to remove subjectivity from scoring and to relieve the time burden of a reporting pathologist. The value of obtaining such important geospatial information using multiplexing of tissue sections was recently demonstrated in a meta-analysis, where mIF/IHC was found to be superior to mRNA expression profiling, tumour mutational burden or methylation data in prediction of patient response to immunotherapy [41]. Therefore, improving patient selection in routine practice may ultimately involve digital image analysis with multiplexed IF or IHC, as recently suggested for nonsmall cell lung cancer [42].

The main shortcoming of our study is that our analysis was based on TMA cores rather than whole slide images (WSI)/tissue sections, which, although common for research assessment, does not allow for assessment of heterogeneity of the TILs infiltrate, especially for B-cells which tend to form lymphoid aggregates. Several studies investigated the number of TMA cores required for accurate representation of whole slide TILs assessment in breast cancer, with one study finding that 4 × 0.6 mm cores (=1.12 mm^2^) was adequate for accurate TILs assessment [43], which compares favourably with our study containing 3 × 1 mm (=2.35 mm^2^), whereas another study suggested that a single 2 mm core from a tumour had 98.9% accuracy in representing the TILs population of the tumour [44]. A comparison of sTILs H/E scoring on WSI and TMA assessment showed only a moderate correlation (Spearman 0.56), with higher levels present in WSI [45] and higher values for specific immune subpopulations on WSI. They also found that human observers tended to score higher numbers of immune subtypes compared to automated image analysis. One recent study also found a slight overestimation of cell counts by image analysis compared to manual counting but no real difference in density [46], whereas good correlation of image analysis with manual counting was found in another study with 96.8% concordance [42]. One large study assessed cell counts on only one single 0.6 mm core for CD8 immunostains [23]. 

It is also important to consider that variations in chemotherapy with contemporary regimes may impact upon the overall local control rate but are generally smaller in quantum than the effect of radiotherapy and would not be expected to change the relative effect of radiotherapy. Similarly, some different phenotypes (particularly luminal A) show less chemotherapy effects and overall benefit of chemotherapy may be greater in luminal B and other phenotypes, but the relative effect of changes seen by TILs should still apply to more modern regimens, including taxanes and the addition of other targeted therapies such as Trastuzumab, even if the absolute quantum is smaller.

## 5. Conclusions

This study highlights the prognostic value of improved characterisation of TILs immunophenotype, which can add to clinical decision-making, especially in TILs low-luminal tumours, for those patients treated with adjuvant radiotherapy. Further studies in larger cohorts with standardised approaches to assessment of cell densities are required. 

## Figures and Tables

**Table 1:** Univariate and multivariate analyses for local recurrence in the whole cohort.

Variables	Univariate				Multivariable (*n* = 456)		
	*n*	HR	95% CI	*p*	HR	95% CI	*p*
CD3 (low vs. high)	484	1.53	0.91–2.55	0.108			
CD8 (low vs. high)	458	1.97	1.16–3.33	0.011	2.34	1.36–4.02	0.002
CD20 (low vs. high)	401	0.90	0.52–1.55	0.70			
CD68 (low vs. high)	464	1.14	0.69–1.90	0.61			
FoxP3 (low vs. high)	446	1.28	0.75–2.18	0.37			
PD1 (low vs. high)	464	1.28	0.76–2.16	0.35			
PDL1 (low vs. high)	415	1.27	0.73–2.21	0.40			
TIL (low vs. high)	484	1.45	0.86–2.42	0.16			
Age (≤50 vs. >50)	485	1.89	1.11–3.21	0.019			
Lymph node (neg vs. pos)	485	0.62	0.37–1.04	0.071			
Endocrine therapy (no vs. yes)	484	1.67	0.98–2.86	0.061			
Histological grade (1–2 vs. 3)	483	0.55	0.32–0.92	0.023	0.48	0.29–0.82	0.007
HER2 status (neg vs. pos)	485	1.12	0.45–2.79	0.81			
Lymphovascular invasion (neg vs. pos)	485	0.95	0.48–1.88	0.89			
Tumour size mm (<20 vs. ≥20)	484	0.70	0.42–1.16	0.16			
Margin (involved vs. clear)	484	3.61	1.30–10.00	0.013	3.71	1.33–10.35	0.012
Radiotherapy Boost (yes vs. no)	485	2.53	1.45–4.39	0.001	2.74	1.56–4.82	<0.001
Chemotherapy (no vs. yes)	485	0.72	0.41–1.25	0.24			

**Table 2:** Univariate and multivariate analyses for overall survival (OS) in the whole cohort.

Variables	Univariate				Multivariable (*n* = 401)		
	*n*	HR	95% CI	*p*	HR	95% CI	*p*
CD3 (low vs. high)	484	1.31	0.97–1.76	0.075			
CD8 (low vs. high)	458	1.56	1.15–2.12	0.005			
CD20 (low vs. high)	401	1.93	1.37–2.71	<0.001	1.73	1.22–2.44	0.002
CD68 (low vs. high)	464	1.12	0.83–1.51	0.46			
FoxP3 (low vs. high)	446	1.41	1.03–1.92	0.030			
PD1 (low vs. high)	464	1.57	1.16–2.14	0.004			
PDL1 (low vs. high)	415	1.12	0.81–1.54	0.50			
TIL (low vs. high)	484	1.35	1.00–1.82	0.046			
Age (≤50 vs. >50)	485	0.48	0.31–0.75	0.001	0.49	0.30–0.79	0.004
Lymph node (neg vs. pos)	485	0.65	0.48–0.88	0.005	0.68	0.48–0.96	0.027
Endocrine therapy (no vs. yes)	484	0.86	0.63–1.14	0.29			
Histological grade (1–2 vs. 3)	483	1.01	0.73–1.40	0.94			
HER2 status (neg vs. pos)	485	1.35	0.71–2.56	0.36			
Lymphovascular invasion (neg vs. pos)	485	0.79	0.54–1.15	0.22			
Tumour size mm (<20 vs. ≥20)	484	0.78	0.57–1.05	0.096			
Chemotherapy (no vs. yes)	485	1.40	0.95–2.05	0.087			

## Data Availability

The datasets generated during and/or analysed during the current study are not publicly available due to ethical constraints but are available from the corresponding author on reasonable request.

## Supplementary Materials

The following are available online at https://www.mdpi.com/2072-6694/12/9/2365/s1: Figure S1: Heatmap of the NanoString BC360 signatures, Table S1: Patient baseline characteristics and treatment details, Table S2: *p* values for association of immune markers with intrinsic subtypes (Kruskal–Wallis (KW) test/Mann–Whitney), Table S3: Association of immune markers with clinicopathological variables, Table S4: Univariate and multivariate analyses for local recurrence in luminal tumours only, Table S5: Univariate and multivariate analyses for local recurrence in triple-negative tumours only, Table S6: Univariate and multivariate analyses for overall survival in luminal tumours only, Table S7: Univariate and multivariate analyses for overall survival in triple-negative tumours only, Table S8: Univariate and multivariate analyses for disease-free survival in the whole cohort (118 events), Table S9: Univariate and multivariate analyses for disease-free survival in luminal A and luminal B (95 events), Table S10: Univariate and multivariate analyses for disease-free survival in triple-negative (21 events), File S1: Opal Multiplex protocol.
