# Supplementary material for: TILs Immunophenotype in Breast Cancer Predicts Local Failure and Overall Survival: Analysis in a Large Radiotherapy Trial with Long-Term Follow-Up
Source: Cancers (Basel). 2020 Aug 21;12(9):2365. doi: 10.3390/cancers12092365 (PMC7563743; doi:10.3390/cancers12092365)
Supplement: Supplementary file 1 [file cancers-12-02365-s001.zip › cancers-881862 File S1.docx]

Supplementary Materials:

TILs immunophenotype in breast cancer predicts local failure and overall survival: analysis in a large cohort with long-term follow-up

Ewan KA Millar, Lois Browne, Iveta Slapetova, Fei Shang, Yuqi Ren, Rachel Bradshaw, Heather Ann Brauer, Sandra O’Toole, Julia Beretov, Renee Whan, Peter H Graham

Opal Multiplex protocol, Manual (Panel 1/Panel 2)

1. Dewax and bring slides from xylene to ethanol to distilled water.
2. Fix sections in 10% neutral buffered formalin (NBF) for 20 minutes.
3. Rinse slides with distilled water and then with antigen retrieval (AR) buffer.
4. Antigen retrieval (AR6), 110^o^C, 5min.
5. Rinse in water and then in TBS.
6. Block with 0.1M Glycine in TBS for 30min.
7. Rinse slides in TBS.
8. Block with Opal antibody diluent/block for 10minutes at RT.
9. Remove blocking solution and add PanCK, 1:2000 (PerkinElmer, OP7LT4001K), incubate for 30 minutes at RT.
10. Rinse slides in TSB 3x2 minutes.
11. Add Polymer HRP Ms+Rb on slides and incubate for 10 minutes at RT.
12. Rinse slides in TSB 3x2 minutes.
13. Add Opal Fluorophone working solution (540, 1:100) on slides and incubate for 10 minutes at RT.
14. Rinse slides in TBS 3x2 minutes.
15. Rinse slides with AR9 buffer.
16. Place slides in processing jar and fill it completely with AR9 buffer, loosely cover the jar with the lid.
17. Microwave 45 seconds at 100% power.
18. Microwave 15 minutes at 20% power.
19. Allow slides cool down for 20 minutes at RT.
20. Rinse with distilled water and then with TBS.
21. Block with Opal antibody diluent/block for 10minutes at RT.
22. Remove blocking solution and add CD3, 1:100 (DAKO, M7254), incubate for 45 minutes at RT.
23. Rinse slides in TSB 3x2 minutes.
24. Add Polymer HRP Ms+Rb on slides and incubate for 10 minutes at RT.
25. Rinse slides in TSB 3x2 minutes.
26. Add Opal Fluorophone working solution (520, 1:100) on slides and incubate for 10 minutes at RT.
27. Rinse slides in TBS 3x2 minutes.
28. Store slides in TBS at 4^o^C overnight.
29. Rinse slides with AR9 buffer.
30. Place slides in processing jar and fill it completely with AR9 buffer, loosely cover the jar with the lid.
31. Microwave 45 seconds at 100% power.
32. Microwave 15 minutes at 20% power.
33. Allow slides cool down for 20 minutes at RT.
34. Rinse with distilled water and then with TBS.
35. Block with Opal antibody diluent/block for 10 minutes at RT.
36. Remove blocking solution and add CD8, 1:150 (PerkinElmer, OP7LT4001K)/PD-L1, 1:25 (Cell Signalling, #13684), incubate for 60 minutes at RT.
37. Rinse slides in TSB 3x2 minutes.
38. Add Polymer HRP Ms+Rb on slides and incubate for 10 minutes at RT.
39. Rinse slides in TSB 3x2 minutes.
40. Add Opal Fluorophone working solution (570, 1:100) on slides and incubate for 10 minutes at RT.
41. Rinse slides in TBS 3x2 minutes.
42. Rinse slides with AR6 buffer.
43. Place slides in processing jar and fill it completely with AR6 buffer, loosely cover the jar with the lid.
44. Microwave 45 seconds at 100% power.
45. Microwave 15 minutes at 20% power.
46. Allow slides cool down for 20 minutes at RT.
47. Rinse with distilled water and then with TBS.
48. Block with Opal antibody diluent/block for 10minutes at RT.
49. Remove blocking solution and add CD20, 1:200 (PerkinElmer, OP7LT4001K)/Foxp3, 1:50 (PerkinElmer, OP7LT4001K), incubate for 45 minutes at RT.
50. Rinse slides in TSB 3x2 minutes.
51. Add Polymer HRP Ms+Rb on slides and incubate for 10 minutes at RT.
52. Rinse slides in TSB 3x2 minutes.
53. Add Opal Fluorophone working solution (620, 1:100) on slides and incubate for 10 minutes at RT.
54. Rinse slides in TBS 3x2 minutes.
55. Rinse slides with AR6 buffer.
56. Microwave treatment, place slides in processing jar and fill it completely with AR6 buffer, loosely cover the jar with the lid.
57. Microwave 45 seconds at 100% power.
58. Microwave 15 minutes at 20% power.
59. Allow slides cool down for 20 minutes at RT.
60. Rinse with distilled water and then with TBS.
61. Block with Opal antibody diluent/block for 10minutes at RT.
62. Remove blocking solution and add CD68, 1:200 (PerkinElmer, OP7LT4001K)/PD-1, 1:50 (abcam, ab137132), incubate for 45 minutes at RT.
63. Rinse slides in TSB 3x2 minutes.
64. Add Polymer HRP Ms+Rb on slides and incubate for 10 minutes at RT.
65. Rinse slides in TSB 3x2 minutes.
66. Add Opal Fluorophone working solution (650, 1:100) on slides and incubate for 10 minutes at RT.
67. Rinse slides in TBS 3x2 minutes.
68. Store slides in TBS at 4^o^C overnight
69. Rinse slides with AR6 buffer.
70. Microwave treatment, place slides in processing jar and fill it completely with AR6 buffer, loosely cover the jar with the lid.
71. Microwave 45 seconds at 100% power.
72. Microwave 15 minutes at 20% power.
73. Allow slides cool down for 20 minutes at RT.
74. Rinse with distilled water and then with TBS.
75. Add DAPI working solution and incubate for 5 minutes at RT.
76. Rinse slides with TBST for 2 minutes and then with distilled water for 2 minutes.
77. Cover slip with prolong antifade mounting medium.
