# Supplementary material for: TILs Immunophenotype in Breast Cancer Predicts Local Failure and Overall Survival: Analysis in a Large Radiotherapy Trial with Long-Term Follow-Up
Source: Cancers (Basel). 2020 Aug 21;12(9):2365. doi: 10.3390/cancers12092365 (PMC7563743; doi:10.3390/cancers12092365)
Supplement: Supplementary file 1 [file cancers-12-02365-s001.zip › cancers-881862 proofed supplementary.docx]

Supplementary Materials:

TILs immunophenotype in breast cancer predicts local failure and overall survival: analysis in a large cohort with long-term follow-up

Ewan KA Millar, Lois Browne, Iveta Slapetova, Fei Shang, Yuqi Ren, Rachel Bradshaw, Heather Ann Brauer, Sandra O’Toole, Julia Beretov, Renee Whan, Peter H Graham


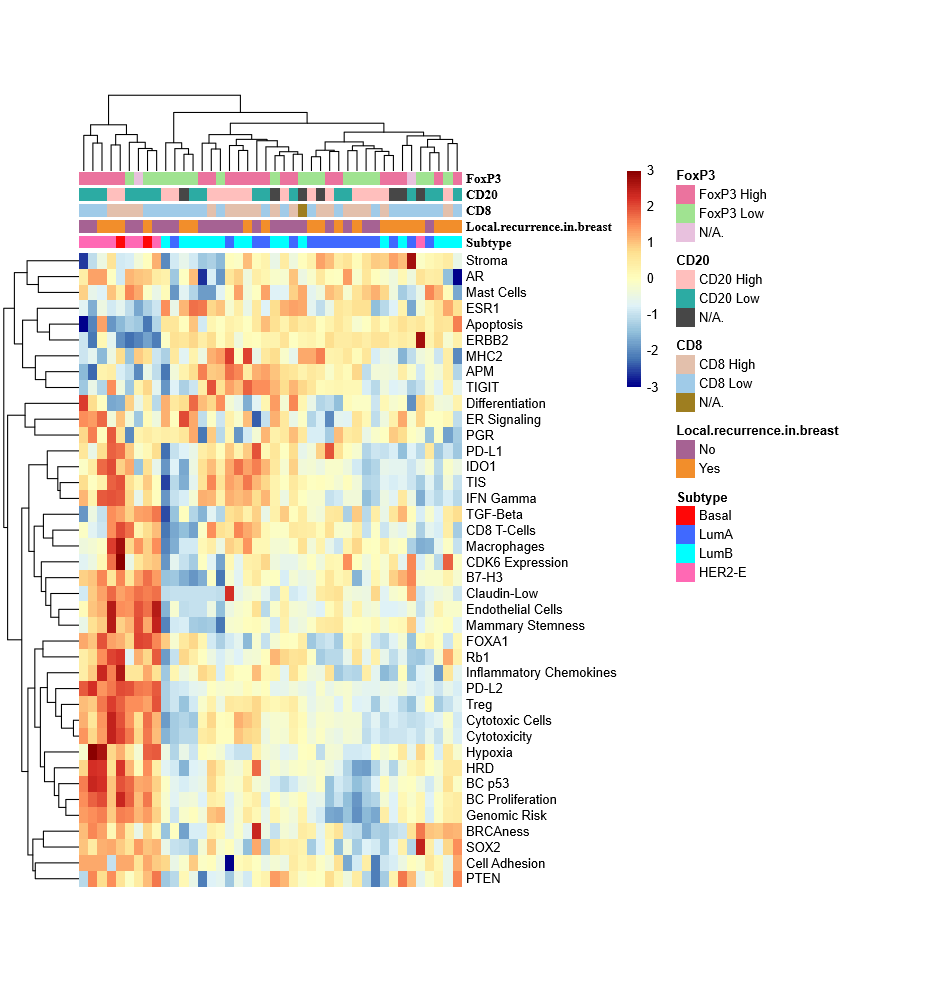


**Figure S1.** Heatmap of the NanoString BC360 signatures.

**Table S1.** Patient baseline characteristics and treatment details.

| Characteristic | No of patients (%) |
| --- | --- |
| Age at diagnosis (years)  (range 24–84)  ≤50  >50 | 102 (21)  383 (79) |
| Tumor size (mM)  T1a (1–5)  T1b (6–10)  T1c (11–19)  T2 (20–50)  T3 (>50) | 5 (1)  73 (15)  227 (47)  178 (37)  1 (<1) |
| Tumor Grade  1  2  3 | 157 (33)  181 (37)  145 (30) |
| Histological type  Ductal, NOS  Lobular  Mucinous  Micropapillary  Metaplastic  Other (apocrine) | 417 (86)  42 (9)  15 (3)  7 (1)  3 (<1)  1 (<1) |
| Lymphnode metastases  N0  N1 (1–3)  N2 (4–10)  N3 (>10) | 146 (30)  339 (70)  127 (26)  17 (4)  2 (<1) |
| ER+ | 406 (84) |
| PR+ | 361 (74) |
| HER-2 amplified (FISH) | 36 (7) |
| Luminal A  Luminal B  Triple negative  HER-2 | 309 (64)  96 (20)  67 (14)  13 (2) |
| Margin - positive | 13 (3) |
| Cavity boost positive  Cavity boost negative | 241 (50)  244 (50) |
| Endocrine therapy | 218 (45) |
| Chemotherapy | 114 (24) |
| Endocrine & chemotherapy | 45 (9) |

**Table S2.** *p* values for association of immune markers with intrinsic subtypes (Kruskal-Wallis (KW) test/Mann Whitney).

| Marker | KW overall | LA- LB | LA- HER2 | LA-TN | LB-HER2 | LB-TN | TN-HER2 |
| --- | --- | --- | --- | --- | --- | --- | --- |
| CD3 | 0.026 | 0.16 | 0.073 | 0.015 | 0.22 | 0.23 | 0.56 |
| CD8 | 0.017 | 0.075 | 0.012 | 0.081 | 0.077 | 0.69 | 0.17 |
| CD20 | 0.006 | 0.016 | 0.069 | 0.010 | 0.41 | 0.71 | 0.53 |
| CD68 | <0.001 | 0.001 | 0.011 | <0.001 | 0.35 | 0.056 | 0.77 |
| FoxP3 | <0.001 | 0.001 | 0.021 | 0.003 | 0.37 | 0.97 | 0.42 |
| PD1 | <0.001 | <0.001 | 0.009 | <0.001 | 0.35 | 0.090 | 0.84 |
| PDL1 | <0.001 | <0.001 | 0.002 | <0.001 | 0.18 | 0.008 | 0.83 |

**Table S3A.** Association of immune markers with clinico-pathological variables.

| Variable |  | | CD3 | | | | | | CD8 | | | | | | CD20 | | | | | | CD68 | | | | | |  |
| --- | --- | --- | --- | --- | --- | --- | --- | --- | --- | --- | --- | --- | --- | --- | --- | --- | --- | --- | --- | --- | --- | --- | --- | --- | --- | --- | --- |
|  | All | | low | | high | | Χ^2^  *p* value | | low | | high | | Χ^2^  *p* value | | low | | high | | Χ^2^  *p* value | | low | | high | | Χ^2^  *p* value | |  |
| **Age**  **<50**  **≥50** | | 102(21)  383(79) | | 37(15)  205(85) | | 65(27)  177(73) | | 9.7  0.002 | | 41(18)  188(82) | | 59(26)  170(74) | | 4.1  0.042 | | 35(17)  169(83) | | 57(29)  140(71) | | 7.9  0.005 | | 44(19)  188(81) | | 57(25)  175(75) | | 2.1  0.144 | |
| **Size**  **<20mm**  **≥20mm** | | 305(63)  179(37) | | 148(61)  93(39) | | 156(64)  86(36) | | 0.48  0.487 | | 141(62)  88(38) | | 143(63)  85(37) | | 0.06  0.800 | | 137(67)  67(33) | | 118(60)  78(40) | | 2.1  0.148 | | 148(64)  84(36) | | 141(61)  90(39) | | 0.37  0.541 | |
| **LVI**  **Present**  **Absent** | | 86(18)  399(82) | | 39(16)  203(84) | | 47(19)  195(81) | | 0.91  0.341 | | 41(18)  188(82) | | 44(19)  185(81) | | 0.13  0.718 | | 32(16)  172(84) | | 39(20)  158(80) | | 1.16  0.281 | | 35(15)  197(85) | | 50(22)  182(78) | | 3.24  0.072 | |
| **Grade**  **1 & 2**  **3** | | 338(70)  145(30) | | 187(78)  53(22) | | 150(62)  92(38) | | 14.5  <0.001 | | 169(74)  60(26) | | 145(64)  82(36) | | 5.23  0.022 | | 156(77)  47(23) | | 116(59)  80(41) | | 14.3  <0.001 | | 182(78)  50(22) | | 135(59)  95(41) | | 21.0  <0.001 | |
| **LN status**  **Pos**  **neg** | | 146(30)  339(70) | | 74(31)  168(69) | | 72(30)  170(70) | | 0.04  0.842 | | 77(34)  152(66) | | 66(29)  163(71) | | 1.23  0.267 | | 63(31)  141(69) | | 54(27)  143(73) | | 0.58  0.445 | | 66(28)  166(72) | | 77(33)  155(67) | | 1.22  0.269 | |
| **ER**  **Pos**  **Neg** | | 406(84)  79(16) | | 206(85)  36(15) | | 199(82)  43(18) | | 0.74  0.389 | | 194(85)  35(15) | | 187(82)  42(18) | | 0.77  0.382 | | 175(86)  29(14) | | 154(78)  43(22) | | 3.94  0.047 | | 203(88)  29(12) | | 183(79)  49(21) | | 6.16  0.013 | |
| **PR**  **Pos**  **Neg** | | 361(74)  124(26) | | 191(79)  51(21) | | 169(70)  73(30) | | 5.25  0.022 | | 170(74)  59(26) | | 167(73)  62(27) | | 0.10  0.751 | | 151(74)  53(26) | | 137(70)  60(30) | | 0.99  0.319 | | 176(76)  56(24) | | 165(71)  67(29) | | 1.34  0.247 | |
| **HER2**  **Pos**  **Neg** | | 36(7)  449(93) | | 12(5)  230(95) | | 24(10)  218(90) | | 4.32  0.038 | | 15(7)  214(93) | | 21(9)  208(91) | | 1.09  0.298 | | 11(5)  193(95) | | 23(12)  174(88) | | 5.10  0.024 | | 17(7)  215(93) | | 19(8)  213(92) | | 0.12  0.729 | |

**Tables S3B.** Association of immune markers with clinico-pathological variables.

| Variable |  | | | TIL cut at median | | | | | | FoxP3 | | | | | | PD-1 | | | | | | PD-L1 | | | | | |
| --- | --- | --- | --- | --- | --- | --- | --- | --- | --- | --- | --- | --- | --- | --- | --- | --- | --- | --- | --- | --- | --- | --- | --- | --- | --- | --- | --- |
|  | All | | low | | high | | Χ^2^  p value | | low | | high | | Χ^2^  p value | | low | | high | | Χ^2^  p value | | low | | high | | Χ^2^  p value | |  |
| **Age**  **<50**  **≥50** | | 102(21)  383(79) | | 37(15)  209(85) | | 65(27)  173(73) | | 10.9  0.001 | | 39(17)  185(83) | | 57(26)  165(74) | | 4.5  0.034 | | 38(16)  194(84) | | 64(28)  168(72) | | 8.5  0.004 | | 31(15)  178(85) | | 60(29)  146(71) | | 12.4  <0.001 | |
| **Size**  **<20mm**  **≥20mm** | | 305(63)  179(37) | | 165(67)  81(33) | | 139(59)  98(41) | | 3.67  0.055 | | 138(62)  86(38) | | 146(66)  75(34) | | 0.96  0.328 | | 145(63)  87(37) | | 147(64)  84(36) | | 0.06  0.800 | | 135(65)  74(35) | | 123(60)  82(40) | | 0.93  0.335 | |
| **LVI**  **Present**  **Absent** | | 86(18)  399(82) | | 39(16)  207(84) | | 47(20)  191(80) | | 1.26  0.263 | | 36(16)  188(84) | | 43(19)  179(81) | | 0.83  0.362 | | 40(17)  192(83) | | 43(19)  189(81) | | 0.13  0.716 | | 37(18)  172(82) | | 41(20)  165(80) | | 0.32  0.566 | |
| **Grade**  **1 & 2**  **3** | | 338(70)  145(30) | | 201(82)  44(18) | | 137(58)  100(42) | | 33.8  <0.001 | | 170(76)  54(24) | | 134(61)  86(39) | | 11.5  0.001 | | 188(81)  44(19) | | 132(57)  98(43) | | 30.3  <0.001 | | 174(83)  35(17) | | 110(54)  94(46) | | 41.4  <0.001 | |
| **LN status**  **Pos**  **neg** | | 146(30)  339(70) | | 71(29)  175(71) | | 75(32)  163(68) | | 0.404  0.525 | | 68(30)  156(70) | | 68(31)  154(69) | | 0.004  0.950 | | 70(30)  162(70) | | 73(31)  159(69) | | 0.09  0763 | | 65(31)  144(69) | | 62(30)  144(70) | | 0.05  0.824 | |
| **ER**  **Pos**  **Neg** | | 406(84)  79(16) | | 223(91)  23(9) | | 183(77)  55(23) | | 16.9  <0.001 | | 197(88)  27(12) | | 175(79)  47(21) | | 6.7  0.010 | | 212(91)  20(9) | | 176(76)  56(24) | | 20.4  <0.001 | | 188(90)  21(10) | | 155(75)  51(25) | | 15.65  <0.001 | |
| **PR**  **Pos**  **Neg** | | 361(74)  124(26) | | 197(80)  49(20) | | 164(69)  74(31) | | 7.97  0.005 | | 164(73)  60(27) | | 166(75)  56(25) | | 0.14  0.707 | | 193(83)  39(17) | | 155(67)  77(33) | | 16.6  <0.001 | | 171(82)  38(18) | | 138(67)  68(33) | | 11.99  0.001 | |
| **HER2**  **Pos**  **Neg** | | 36(7)  449(93) | | 9(4)  237(96) | | 27(11)  211(89) | | 10.4  0.001 | | 11(5)  213(95) | | 25(11)  197(89) | | 6.06  0.014 | | 12(5)  220(95) | | 24(10)  208(90) | | 4.34  0.037 | | 8(4)  201(96) | | 22(11)  184(89) | | 7.26  0.007 | |

**Table S4.** Univariate and multivariate analyses for local recurrence in Luminal tumours only.

| Variables | Univariate | | | | Multivariable (*n* = 379) | | |
| --- | --- | --- | --- | --- | --- | --- | --- |
|  | n | HR | 95%CI | p | HR | 95%CI | p |
| CD3 (low vs high) | 404 | 1.51 | 0.86-2.66 | 0.15 |  |  |  |
| CD8 (low vs high ) | 380 | 1.91 | 1.07-3.39 | 0.028 | 2.19 | 1.23-3.92 | 0.008 |
| CD20 (low vs high) | 327 | 0.82 | 0.45-1.50 | 0.52 |  |  |  |
| CD68 (low vs high) | 385 | 1.17 | 0.67-2.03 | 0.59 |  |  |  |
| FoxP3 (low vs high) | 370 | 1.04 | 0.58-1.87 | 0.90 |  |  |  |
| PD1 (low vs high) | 388 | 1.08 | 0.62-1.90 | 0.79 |  |  |  |
| PDL1 (low vs high) | 344 | 1.21 | 0.66-2.23 | 0.53 |  |  |  |
| TIL (low vs high) | 405 | 1.61 | 0.91-2.87 | 0.10 |  |  |  |
| Age (≤50 vs >50) | 405 | 2.74 | 1.55-4.84 | 0.001 | 2.85 | 1.60-5.07 | <0.001 |
| Lymph node (neg vs pos) | 405 | 0.68 | 0.38-1.19 | 0.18 |  |  |  |
| Endocrine therapy (no vs yes) | 404 | 1.65 | 0.94-2.89 | 0.082 |  |  |  |
| Histological grade ( 1-2 vs 3) | 403 | 0.46 | 0.25-0.82 | 0.009 |  |  |  |
| HER2 status (neg vs pos) | 405 | 1.53 | 0.55-4.24 | 0.42 |  |  |  |
| Lymphovascular invasion (neg vs pos) | 405 | 1.07 | 0.50-2.28 | 0.86 |  |  |  |
| Tumour size mm (<20 vs $\geq$20) | 404 | 0.76 | 0.43-1.35 | 0.35 |  |  |  |
| Margin (involved vs clear) | 404 | 3.79 | 1.36-10.58 | 0.011 | 3.52 | 1.26-9.82 | 0.016 |
| Radiotherapy Boost (y vs no) | 405 | 2.10 | 1.17-3.76 | 0.013 | 2.21 | 1.23-3.96 | 0.008 |
| Chemotherapy (no vs yes) | 405 | 0.59 | 0.31-1.12 | 0.11 |  |  |  |

HR Hazard ratio by Cox proportional hazard model.

**Table S5.** Univariate and multivariate analyses for local recurrence in triple negative tumours only.

| Variables | Univariate | | | | Multivariable (*n* = 67) | | |
| --- | --- | --- | --- | --- | --- | --- | --- |
|  | n | HR | 95%CI | p | HR | 95%CI | p |
| CD3 (low vs high) | 67 | 0.93 | 0.22-3.89 | 0.92 |  |  |  |
| CD8 (low vs high) | 65 | 2.35 | 0.56-9.86 | 0.24 |  |  |  |
| CD20 (low vs high) | 61 | 0.92 | 0.22-3.84 | 0.91 |  |  |  |
| CD68 (low vs high) | 66 | 1.18 | 0.28-4.96 | 0.82 |  |  |  |
| FoxP3 (low vs high) | 63 | 2.56 | 0.60-10.87 | 0.20 |  |  |  |
| PD1 (low vs high) | 63 | 2.22 | 0.49-10.14 | 0.30 |  |  |  |
| PDL1 (low vs high) | 60 | 1.33 | 0.24-7.28 | 0.74 |  |  |  |
| TIL (low vs high) | 66 | 0.87 | 0.22-3.46 | 0.84 |  |  |  |
| Age (≤50 vs >50) | 67 | 0.52 | 0.11-2.60 | 0.43 |  |  |  |
| Lymph node (neg vs pos) | 67 | 0.38 | 0.09-1.59 | 0.18 |  |  |  |
| Histological grade ( 1-2 vs 3) | 67 | 0.59 | 0.07-4.76 | 0.62 |  |  |  |
| Lymphovascular invasion (neg vs pos) | 67 | 0.46 | 0.09-2.28 | 0.34 |  |  |  |
| Tumour size mm (<20 vs $\geq$20) | 67 | 0.35 | 0.07-1.73 | 0.20 |  |  |  |
| Omit |  |  |  |  |  |  |  |
| Radiotherapy Boost (y vs no) | 67 | 9.94 | 1.22-80.91 | 0.032 | 9.94 | 1.22-80.91 | 0.032 |
| Chemotherapy (no vs yes) | 67 | 0.92 | 0.22-3.84 | 0.91 |  |  |  |

HR Hazard ratio by Cox proportional hazard model.

**Table 6.** Univariate and multivariate analyses for overall survival in luminal tumours only.

| Variables | Univariate | | | | Multivariable (*n* = 327) | | |
| --- | --- | --- | --- | --- | --- | --- | --- |
|  | n | HR | 95%CI | p | HR | 95%CI | p |
| CD3 (low vs high) | 404 | 1.15 | 0.84-1.60 | 0.38 |  |  |  |
| CD8 (low vs high) | 380 | 1.53 | 1.09-2.15 | 0.013 |  |  |  |
| CD20 (low vs high) | 327 | 1.75 | 1.20-2.55 | 0.004 | 1.67 | 1.14-2.44 | 0.008 |
| CD68 (low vs high) | 385 | 1.14 | 0.82-1.59 | 0.44 |  |  |  |
| FoxP3 (low vs high) | 370 | 1.24 | 0.88-1.74 | 0.21 |  |  |  |
| PD1 (low vs high) | 388 | 1.40 | 1.00-1.97 | 0.049 |  |  |  |
| PDL1 (low vs high) | 344 | 0.94 | 0.66-1.34 | 0.73 |  |  |  |
| TIL (low vs high) | 405 | 1.34 | 0.96-1.86 | 0.081 |  |  |  |
| Age (≤50 vs >50) | 405 | 0.51 | 0.30-0.85 | 0.010 | 0.48 | 0.27-0.87 | 0.014 |
| Lymph node (neg vs pos) | 405 | 0.71 | 0.51-0.99 | 0.045 |  |  |  |
| Endocrine therapy (no vs yes) | 404 | 0.83 | 0.60-1.15 | 0.26 |  |  |  |
| Histological grade ( 1-2 vs 3) | 403 | 1.00 | 0.66-1.50 | 0.99 |  |  |  |
| HER2 status (neg vs pos) | 405 | 1.01 | 0.50-2.06 | 0.98 |  |  |  |
| Lymphovascular invasion (neg vs pos) | 405 | 0.85 | 0.57-1.29 | 0.45 |  |  |  |
| Tumour size mm (<20 vs $\geq$20) | 404 | 0.71 | 0.51-0.98 | 0.040 |  |  |  |
| Chemotherapy ( no vs yes) | 405 | 1.46 | 0.88-2.42 | 0.14 |  |  |  |

HR Hazard ratio by Cox proportional hazard model.

**Table S7.** Univariate and multivariate analyses for overall survival in triple negative tumours only.

| Variables | Univariate | | | | Multivariable (*n* = 60) | | |
| --- | --- | --- | --- | --- | --- | --- | --- |
|  | n | HR | 95%CI | p | HR | 95%CI | p |
| CD3 (low vs high) | 67 | 2.04 | 0.94-4.43 | 0.070 |  |  |  |
| CD8 (low vs high) | 65 | 1.45 | 0.67-3.13 | 0.35 |  |  |  |
| CD20 (low vs high) | 61 | 2.73 | 1.22-6.12 | 0.014 |  |  |  |
| CD68 (low vs high) | 66 | 1.05 | 0.47-2.37 | 0.91 |  |  |  |
| FoxP3 (low vs high) | 63 | 2.37 | 1.07-5.25 | 0.033 |  |  |  |
| PD1 (low vs high) | 63 | 2.50 | 1.15-5.44 | 0.020 |  |  |  |
| PDL1 (low vs high) | 60 | 3.04 | 1.36-6.80 | 0.007 | 3.44 | 1.53-7.73 | 0.003 |
| TIL (low vs high) | 66 | 1.43 | 0.65-3.16 | 0.38 |  |  |  |
| Age (≤50 vs >50) | 67 | 0.46 | 0.19-1.16 | 0.099 |  |  |  |
| Lymph node (neg vs pos) | 67 | 0.31 | 0.14-0.70 | 0.005 | 0.27 | 0.12-0.64 | 0.003 |
| Histological grade ( 1-2 vs 3) | 67 | 1.02 | 0.38-2.69 | 0.98 |  |  |  |
| Lymphovascular invasion (neg vs pos) | 67 | 0.44 | 0.17-1.09 | 0.077 |  |  |  |
| Tumour size mm (<20 vs $\geq$20) | 67 | 1.11 | 0.51-2.40 | 0.80 |  |  |  |
| Chemotherapy ( no vs yes) | 67 | 1.22 | 0.56-2.65 | 0.62 |  |  |  |

HR Hazard ratio by Cox proportional hazard model.

**Table S8.** Univariate and multivariate analyses for disease free survival in the whole cohort (118 events).

| Variables | Univariate | | | | Multivariable (*n* = 456) | | |
| --- | --- | --- | --- | --- | --- | --- | --- |
|  | n | HR | 95%CI | p | HR | 95%CI | p |
| CD3 (low vs high) | 484 | 1.28 | 0.89-1.85 | 0.18 |  |  |  |
| CD8 (low vs high) | 458 | 1.78 | 1.23-2.58 | 0.002 | 1.60 | 1.10-2.33 | 0.015 |
| CD20 (low vs high) | 401 | 1.30 | 0.87-1.92 | 0.19 |  |  |  |
| CD68 (low vs high) | 464 | 0.93 | 0.64-1.33 | 0.67 |  |  |  |
| FoxP3 (low vs high) | 446 | 1.35 | 0.93-1.96 | 0.12 |  |  |  |
| PD1 (low vs high) | 464 | 1.30 | 0.90-1.88 | 0.17 |  |  |  |
| PDL1 (low vs high) | 415 | 1.08 | 0.73-1.59 | 0.71 |  |  |  |
| TIL (low vs high) | 484 | 1.19 | 0.82-1.70 | 0.36 |  |  |  |
| Age (≤50 vs >50) | 485 | 1.55 | 1.04-2.30 | 0.031 |  |  |  |
| Lymph node (neg vs pos) | 485 | 0.38 | 0.26-0.54 | <0.001 | 0.31 | 0.20-0.47 | <0.001 |
| Endocrine therapy (no vs yes) | 484 | 1.28 | 0.89-1.85 | 0.19 | 2.52 | 1.65-3.83 | <0.001 |
| Histological grade ( 1-2 vs 3) | 483 | 0.60 | 0.42-0.87 | 0.007 |  |  |  |
| HER2 status (neg vs pos) | 485 | 0.89 | 0.43-1.82 | 0.75 |  |  |  |
| Lymphovascular invasion (neg vs pos) | 485 | 0.50 | 0.33-0.75 | 0.001 |  |  |  |
| Tumour size mm (<20 vs $\geq$20) | 484 | 0.51 | 0.36-0.73 | <0.001 | 0.58 | 0.40-0.85 | 0.005 |
| Margin (involved vs clear) | 484 | 2.43 | 1.07-5.53 | 0.035 | 2.31 | 1.01-5.30 | 0.048 |
| Radiotherapy Boost (y vs no) | 485 | 1.28 | 0.89-1.84 | 0.19 |  |  |  |
| Chemotherapy (no vs yes) | 485 | 0.58 | 0.39-0.84 | 0.004 |  |  |  |

**Table S9.** Univariate and multivariate analyses for disease free survival in Luminal A & Luminal B (95 events).

| Variables | Univariate | | | | Multivariable (*n* = 380) | | |
| --- | --- | --- | --- | --- | --- | --- | --- |
|  | n | HR | 95%CI | p | HR | 95%CI | p |
| CD3 (low vs high) | 404 | 1.20 | 0.80-1.80 | 0.38 |  |  |  |
| CD8 (low vs high) | 380 | 1.74 | 1.15-2.65 | 0.009 | 1.73 | 1.13-2.63 | 0.011 |
| CD20 (low vs high) | 327 | 1.13 | 0.73-1.76 | 0.58 |  |  |  |
| CD68 (low vs high) | 385 | 0.98 | 0.66-1.48 | 0.94 |  |  |  |
| FoxP3 (low vs high) | 370 | 1.17 | 0.77-1.78 | 0.47 |  |  |  |
| PD1 (low vs high) | 388 | 1.19 | 0.78-1.80 | 0.42 |  |  |  |
| PDL1 (low vs high) | 344 | 0.96 | 0.62-1.48 | 0.84 |  |  |  |
| TIL (low vs high) | 405 | 1.17 | 0.78-1.75 | 0.46 |  |  |  |
| Age (≤50 vs >50) | 405 | 2.07 | 1.34-3.21 | 0.001 | 1.87 | 1.21-2.93 | 0.005 |
| Lymph node (neg vs pos) | 405 | 0.38 | 0.26-0.57 | <0.001 | 0.43 | 0.29-0.65 | <0.001 |
| Endocrine therapy (no vs yes) | 404 | 1.25 | 0.84-1.87 | 0.28 |  |  |  |
| Histological grade ( 1-2 vs 3) | 403 | 0.56 | 0.36-0.88 | 0.011 |  |  |  |
| HER2 status (neg vs pos) | 405 | 1.18 | 0.51-2.69 | 0.70 |  |  |  |
| Lymphovascular invasion (neg vs pos) | 405 | 0.57 | 0.36-0.91 | 0.019 |  |  |  |
| Tumour size mm (<20 vs $\geq$20) | 404 | 0.50 | 0.34-0.75 | 0.001 |  |  |  |
| Margin (involved vs clear) | 404 | 2.68 | 1.17-6.15 | 0.020 |  |  |  |
| Radiotherapy Boost (y vs no) | 405 | 1.03 | 0.69-1.54 | 0.88 |  |  |  |
| Chemotherapy (no vs yes) | 405 | 0.44 | 0.28-0.68 | <0.001 |  |  |  |

**Table S10.** Univariate and multivariate analyses for disease free survival in Triple Negative (21 events).

| Variables | Univariate | | | | Multivariable (*n* = 63) | | |
| --- | --- | --- | --- | --- | --- | --- | --- |
|  | n | HR | 95%CI | p | HR | 95%CI | p |
| CD3 (low vs high) | 67 | 1.35 | 0.57-3.18 | 0.49 |  |  |  |
| CD8 (low vs high) | 65 | 1.83 | 0.77-4.36 | 0.17 |  |  |  |
| CD20 (low vs high) | 61 | 1.90 | 0.79-4.59 | 0.15 |  |  |  |
| CD68 (low vs high) | 66 | 0.78 | 0.30-2.01 | 0.61 |  |  |  |
| FoxP3 (low vs high) | 63 | 2.34 | 0.96-5.66 | 0.060 |  |  |  |
| PD1 (low vs high) | 63 | 1.84 | 0.75-4.52 | 0.19 | 8.56 | 2.33-31.45 | 0.001 |
| PDL1 (low vs high) | 60 | 2.19 | 0.86-5.57 | 0.098 |  |  |  |
| TIL (low vs high) | 66 | 1.20 | 0.50-2.89 | 0.69 |  |  |  |
| Age (≤50 vs >50) | 67 | 0.66 | 0.25-1.70 | 0.39 |  |  |  |
| Lymph node (neg vs pos) | 67 | 0.30 | 0.13-0.72 | 0.007 | 0.23 | 0.06-0.81 | 0.023 |
| Histological grade ( 1-2 vs 3) | 67 | 0.71 | 0.21-2.42 | 0.59 |  |  |  |
| Lymphovascular invasion (neg vs pos) | 67 | 0.22 | 0.09-0.53 | 0.001 | 0.20 | 0.07-0.58 | 0.003 |
| Tumour size mm (<20 vs $\geq$20) | 67 | 0.51 | 0.21-1.27 | 0.15 |  |  |  |
| Radiotherapy Boost (y vs no) | 67 | 3.56 | 1.38-9.19 | 0.009 | 5.19 | 1.72-15.69 | 0.004 |
| Chemotherapy (no vs yes) | 67 | 1.14 | 0.48-2.71 | 0.76 |  |  |  |

Note HER2 not included and margin status not included (all had clear margins).
